# Artificial intelligence for multi-time-point arterial phase contrast-enhanced MRI profiling to predict prognosis after transarterial chemoembolization in hepatocellular carcinoma

**DOI:** 10.1007/s11547-025-02043-6

**Published:** 2025-07-24

**Authors:** Lanlin Yao, Hamzah Adwan, Simon Bernatz, Hao Li, Thomas J. Vogl

**Affiliations:** Clinic for Radiology and Nuclear Medicine, University Hospital Frankfurt, Goethe University, Theodor-Stern-Kai 7, 60590 Frankfurt Am Main, Germany

**Keywords:** Hepatocellular carcinoma, MRI, Artificial intelligence, Swin-transformer, Transarterial chemoembolization

## Abstract

**Purpose:**

Contrast-enhanced magnetic resonance imaging (CE-MRI) monitoring across multiple time points is critical for optimizing hepatocellular carcinoma (HCC) prognosis during transarterial chemoembolization (TACE) treatment. The aim of this retrospective study is to develop and validate an artificial intelligence (AI)-powered models utilizing multi-time-point arterial phase CE-MRI data for HCC prognosis stratification in TACE patients.

**Material and methods:**

A total of 543 individual arterial phase CE-MRI scans from 181 HCC patients were retrospectively collected in this study. All patients underwent TACE and longitudinal arterial phase CE-MRI assessments at three time points: prior to treatment, and following the first and second TACE sessions. Among them, 110 patients received TACE monotherapy, while the remaining 71 patients underwent TACE in combination with microwave ablation (MWA). All images were subjected to standardized preprocessing procedures. We developed an end-to-end deep learning model, ProgSwin-UNETR, based on the Swin Transformer architecture, to perform four-class prognosis stratification directly from input imaging data. The model was trained using multi-time-point arterial phase CE-MRI data and evaluated via fourfold cross-validation. Classification performance was assessed using the area under the receiver operating characteristic curve (AUC). For comparative analysis, we benchmarked performance against traditional radiomics-based classifiers and the mRECIST criteria. Prognostic utility was further assessed using Kaplan–Meier (KM) survival curves. Additionally, multivariate Cox proportional hazards regression was performed as a post hoc analysis to evaluate the independent and complementary prognostic value of the model outputs and clinical variables. GradCAM +  + was applied to visualize the imaging regions contributing most to model prediction.

**Results:**

The ProgSwin-UNETR model achieved an accuracy of 0.86 and an AUC of 0.92 (95% CI: 0.90–0.95) for the four-class prognosis stratification task, outperforming radiomic models across all risk groups. Furthermore, KM survival analyses were performed using three different approaches—AI model, radiomics-based classifiers, and mRECIST criteria—to stratify patients by risk. Of the three approaches, only the AI-based ProgSwin-UNETR model achieved statistically significant risk stratification across the entire cohort and in both TACE-alone and TACE + MWA subgroups (*p* < 0.005). In contrast, the mRECIST and radiomics models did not yield significant survival differences across subgroups (*p* > 0.05). Multivariate Cox regression analysis further demonstrated that the model was a robust independent prognostic factor (*p* = 0.01), effectively stratifying patients into four distinct risk groups (Class 0 to Class 3) with Log(HR) values of 0.97, 0.51, −0.53, and −0.92, respectively. Additionally, GradCAM +  + visualizations highlighted critical regional features contributing to prognosis prediction, providing interpretability of the model.

**Conclusion:**

ProgSwin-UNETR can well predict the various risk groups of HCC patients undergoing TACE therapy and can further be applied for personalized prediction.

## Introduction

Hepatocellular carcinoma (HCC) accounts for 75%–85% of primary liver cancer cases. Due to its increasing incidence and mortality over the past two decades, HCC continues to pose a serious global health challenge [1]. Transarterial chemoembolization (TACE), primarily recommended for intermediate-stage (Barcelona Clinic Liver Cancer, BCLC B) HCC, is a treatment option for patients unsuitable for curative therapies [2, 3]. In clinical practice, however, TACE has also been selectively applied in early (BCLC A) and advanced (BCLC C) stages, often as part of individualized strategies guided by multidisciplinary discussions [4–7]. Despite its efficacy, predicting patient response to TACE remains challenging, as only about half of HCC patients respond to the initial treatment [8, 9]. Among those with insufficient response, many require repeated TACE procedures or additional therapies, such as microwave ablation or systemic treatment. These needs are primarily attributable to tumor heterogeneity and patient-specific biological variability [10–13]. As a result, treatment decisions often rely on expert consensus meetings, yet reliable and objective decision support tools remain lacking.

Recent advances in computational power have significantly enhanced artificial intelligence (AI)-based tools for liver cancer prognosis [14–19]. Traditionally, the modified RECIST (mRECIST) criteria has been widely used to evaluate TACE response [20, 21]. However, mRECIST primarily focuses on measurable target lesions, potentially overlooking treatment responses in non-target lesions and failing to fully capture intralesional heterogeneity. Its accuracy is also limited by inter-radiologist variability [22]. Recent studies indicate that three-dimensional (3D) tumor morphology and dynamic volumetric changes outperform two-dimensional (2D) methods for longitudinal monitoring in solid tumors [23–26]. To address the limitations of traditional imaging criteria in HCC prognosis, deep learning (DL)-based approaches have emerged as powerful alternatives. These methods either extract radiomics features for use in conventional models or employ end-to-end learning to directly capture prognostic signatures from imaging data [14, 27–32]. Among deep learning frameworks, the Transformer—originally developed for natural language processing (NLP) tasks such as ChatGPT [33, 34]—has gained notable traction in medical imaging. Variants such as the Vision Transformer (ViT) and Swin Transformer have achieved state-of-the-art results in multi-phase MRI segmentation [35–38]. The Swin Transformer, in particular, addresses key challenges in 3D medical imaging by hierarchically capturing both global and local features via a sliding window mechanism. It enables precise spatial representation of high-resolution volumetric MRI, facilitating comprehensive analysis of target and non-target lesions before and after TACE. These capabilities support improved quantification of intralesional heterogeneity and treatment response. Building upon this foundation, Swin-UNETR integrates the Swin Transformer backbone with a UNet-inspired decoding pathway, achieving state-of-the-art performance in 3D medical image segmentation. Beyond segmentation, its scalable design is well-suited for downstream applications, such as risk stratification and prognostic modeling [39, 40]. Leveraging this framework has the potential to support personalized, image-guided decision-making in HCC management.

In this study, we implemented an end-to-end deep learning model based on the Swin Transformer architecture for early and accurate risk stratification in HCC patients undergoing TACE—a pressing challenge given the marked variability in therapeutic response. The model uses multi-time-point arterial phase contrast-enhanced MRI to capture dynamic enhancement patterns. These patterns reflect tumor vascularity and internal structural complexity, both of which are correlated with prognosis [41]. To our knowledge, this is the first study to apply the Swin Transformer architecture to longitudinal prognosis modeling in HCC. Our findings suggest that AI-driven imaging analysis may support more personalized treatment decisions and improve outcomes in liver cancer care.

Prognostic stratification in HCC remains a critical unmet need, particularly among patients receiving TACE, where therapeutic response is highly heterogeneous and often difficult to predict. Timely identification of individuals at increased risk of progression or treatment failure could inform clinical decision-making, optimize surveillance strategies, and enable personalized care. In this study, we implemented a deep learning model that processes longitudinal contrast-enhanced MRI to extract comprehensive imaging features from the entire liver. These deep imaging features enabled the model to predict patient prognosis and stratify clinical risk. Our findings suggest that AI-driven imaging analysis may serve as a non-invasive tool to improve risk assessment and guide treatment planning in liver cancer.

## Materials and methods

### Participants and data source

**Fig. 1:**
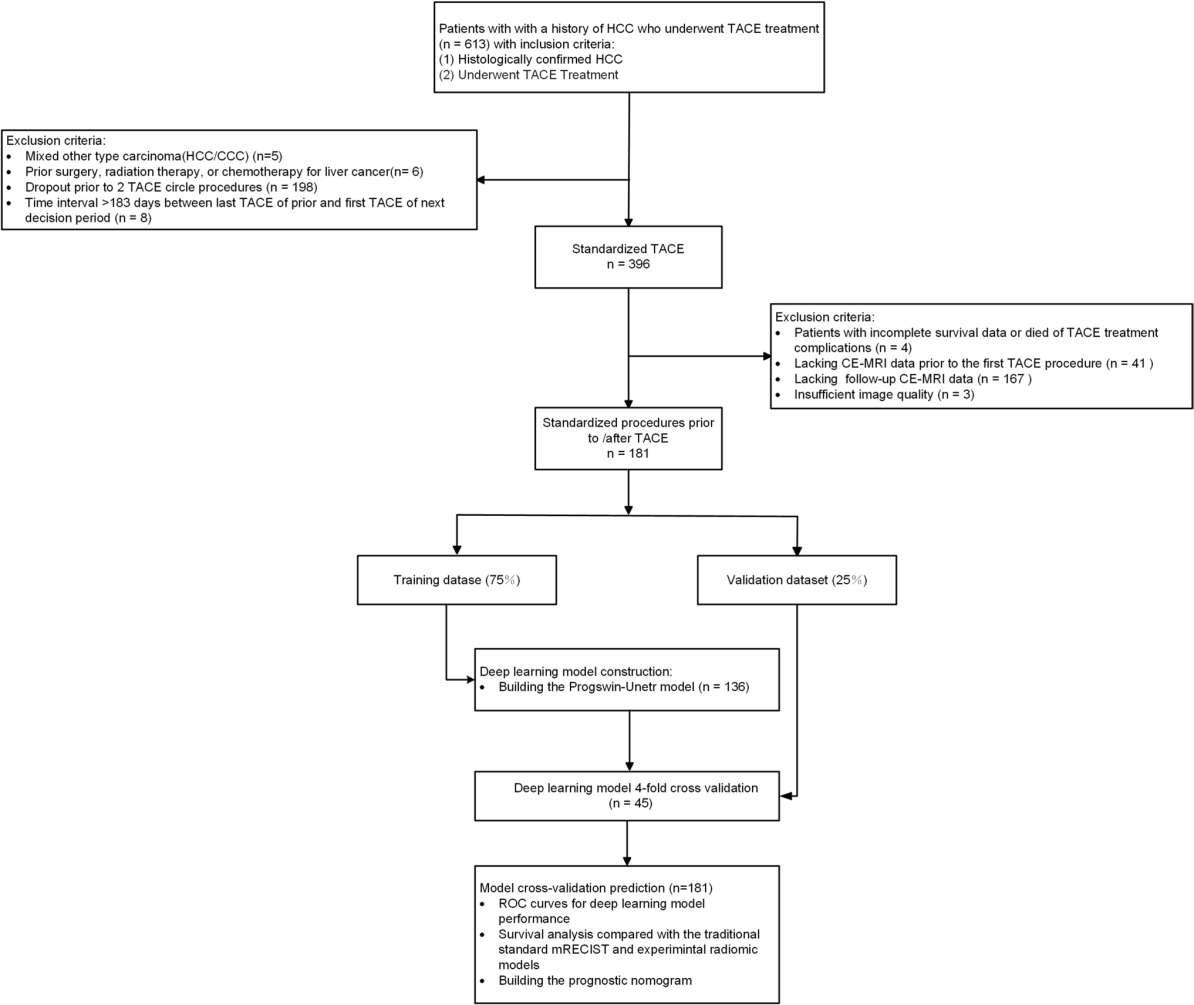
STARD flowchart of inclusion and exclusion of study patients. STARD, standards for reporting diagnostic accuracy studies

This retrospective study was approved by the XXX Ethics Committee, and all patients provided informed consent (project-number: XXX). We collected 181 HCC-patients (male, 151; mean age, 65.6 ± 10.9 years) who were treated with TACE between 01/2012 and 01/2022. Inclusion criteria were: (1) Histologically confirmed HCC, (2) TACE treatment. Exclusion criteria: (1) Diagnosis of mixed-type carcinoma, (2) History of prior hepatic resection or liver transplantation, previous receipt of radiation therapy or chemotherapy for liver cancer before TACE treatment, (3) less than 2 TACE procedures, (4) (4) Time interval > 183 days between last TACE of prior and first TACE of next decision period, (5) Patients with incomplete survival data or died of TACE treatment complications, (6) no arterial phase CE-MRI data prior to the first TACE procedure, (7) no follow-up arterial phase CE-MRI data, (8) insufficient image quality. One hundred eighty-one patients met the criteria and were evaluated. The workflow of the case identification process is depicted in Fig. 1.

All patients underwent TACE treatment. To further explore the application of artificial intelligence (AI) technology, we categorized the TACE cohort into two main subgroups based on the treatment received: patients treated with TACE alone and those treated with TACE combined with MWA. Arterial phase CE-MRI images were acquired before the initial TACE procedure and during follow-up after each treatment session.

### Clinical and therapeutic characteristics

This retrospective study included patients with HCC who received either TACE or combined TACE and MWA. Treatment decisions were made in routine clinical practice, guided primarily by EASL recommendations and finalized through multidisciplinary tumor board (MDT) discussions. The choice of treatment reflected individual patient factors, such as tumor burden, anatomical considerations, liver function, and overall clinical status. TACE monotherapy was generally indicated for patients with multifocal, liver-confined HCC unsuitable for curative resection or ablation. However, in certain cases, TACE monotherapy was performed outside standard guidelines when individualized evaluation determined it to be the most appropriate therapeutic option, particularly in patients with unique anatomical or clinical characteristics. Eligible patients typically exhibited preserved liver function (Child–Pugh class A or well-compensated B7), good performance status (ECOG 0–1), and no evidence of macrovascular invasion or extrahepatic metastasis (technical procedure outlined in Appendix 1). Combined TACE and microwave ablation MWA therapy was indicated for patients where curative intent could potentially be achieved by adjunctive treatment. Selection criteria included: (1) the presence of a dominant tumor nodule amenable to targeted ablation following TACE-induced devascularization; (2) tumor size exceeding conventional ablation thresholds (> 3 cm up to ~ 5 cm); (3) tumors located near major hepatic vessels where pre-ablation devascularization would reduce heat-sink effects; or (4) residual viable tumor remaining after initial TACE. As with TACE monotherapy, selection for combination therapy was individualized based on MDT consensus, reflecting both guideline recommendations and real-world clinical considerations (technical procedure outlined in Appendix 2).

### Imaging protocol

The arterial phase CE-MRI protocol was performed using a dynamic contrast-enhanced MRI system (Magnetom Espree; Magnetom Avanto-fit; Siemens, Erlangen, Germany) for pre-treatment assessment and tumor response evaluation. All patients underwent arterial phase contrast-enhanced MRI (CE-MRI) scans before and after the initial TACE procedure. For contrast-enhanced imaging, 0.1 mmol/kg body weight of either Gadoteric acid (Dotarem®, Guerbet GmbH, Sulzbach, Germany) or Gadobutrol (Gadovist®, Bayer Vital GmbH, Leverkusen, Germany) was administered. Contrast-enhanced imaging was performed prior to the first treatment and 4 weeks after each subsequent TACE session. The standard imaging protocol included the following sequences: unenhanced and contrast-enhanced T1-weighted and T2-weighted MRI scans performed on a 1.5-T or 3-T system with a 5-mm transverse section thickness. The sequences applied were diffusion-transverse, EP-2D-Diff (b50, b400, b800), HASTE, in- and opposed-phase, TSE, FLASH, and contrast-enhanced FLASH dynamic phase imaging. This study focused specifically on the arterial phase, utilizing contrast-enhanced FLASH dynamic phase imaging for tumor assessment.

### AI model construction

**Fig. 2:**
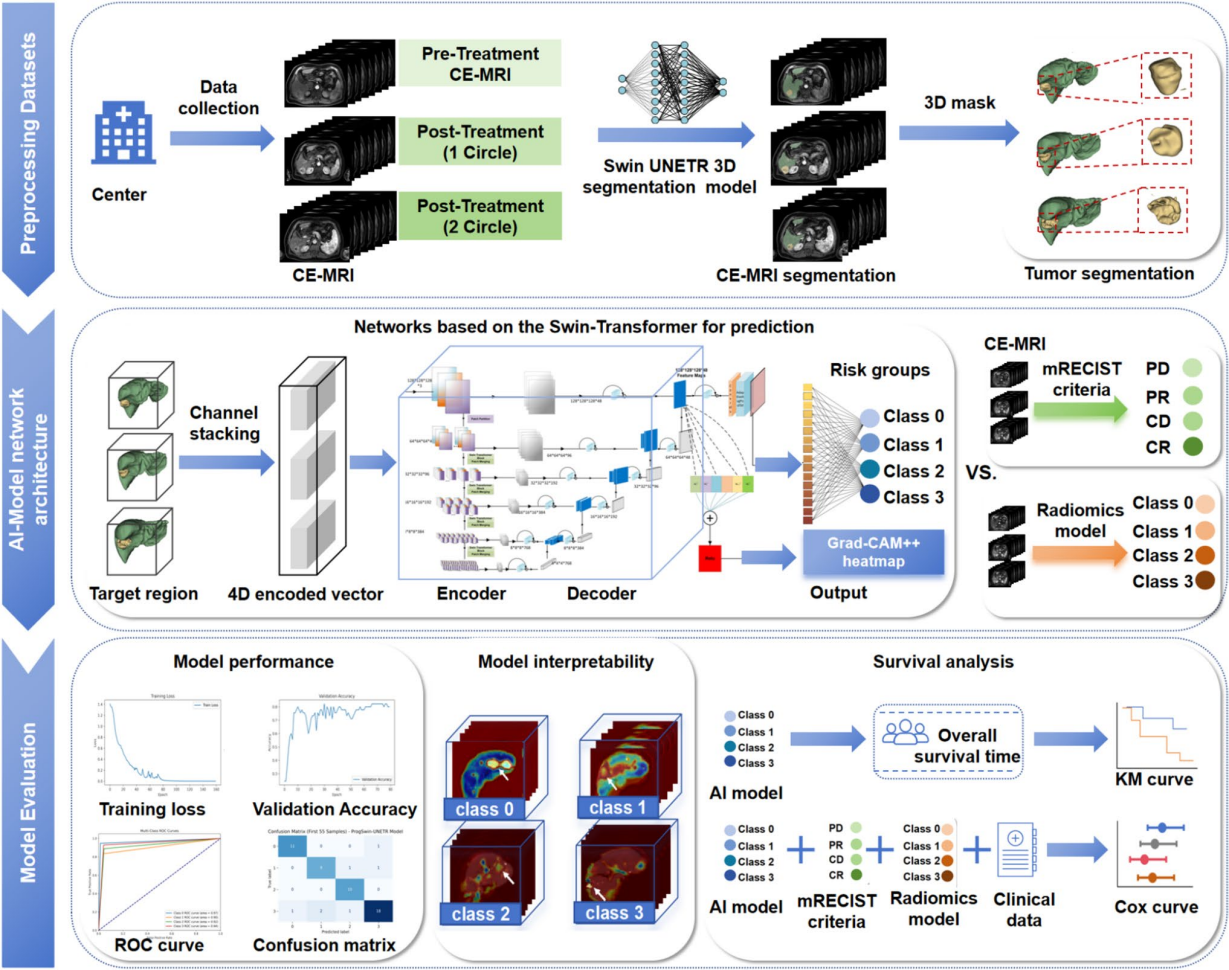
Workflow of the ProgSwin-UNETR Model for HCC Patients. This figure illustrates the comprehensive workflow divided into three main sections: dataset preprocessing, network architecture development, and model evaluation. Initially, the preprocessing step generated segmented images that displayed the masks of liver background and tumor areas at both the baseline and post-treatment phases. These segmented outputs were subsequently transformed into detailed 3D visualizations, offering a vivid and comprehensive view of the anatomical changes over time. Subsequently, the original DBI-MRI datasets, along with the generated 3D masks, were input into the ProgSwin-UNETR model for further analysis and processing. The model was developed based on the proposed Swin-UNETR architecture, which integrated U-Net networks with Swin-Transformer technology. Classification outcomes were determined through an adaptive three-dimensional average pooling layer paired with a ReLU activation function. Its effectiveness was demonstrated through ROC, KM, and Cox curves, which assessed the prognostic predictive ability in comparison with radiomic models and mRECIST criteria

The overall workflow of the proposed ProgSwin-UNETR deep learning framework is illustrated in Fig. 2. This model was developed to perform multi-class prognostic risk stratification for HCC patients based on contrast-enhanced arterial-phase MRI. All input images—acquired at three clinical time points (prior to treatment, and after the first and second TACE sessions)—underwent standardized preprocessing, including automated 3D segmentation of liver and tumor regions using a Swin-UNETR-based algorithm, achieving a Dice coefficient of 0.83 (see Appendix 3). This step reduced background noise and highlighted relevant anatomical structures. We adapted the Swin-UNETR backbone for multi-class prognostic prediction by modifying the input and output layers. The final ProgSwin-UNETR model comprises four components: (1) an image enhancement module that processed 3D volumes guided by segmentation masks, resampled to 128 × 128 × 128 voxels, and stacked into a 4D input tensor across three time points; (2) a Swin Transformer-based encoder to extract spatially informative features via hierarchical self-attention; (3) a UNet-inspired decoder repurposed for classification, aggregating multiscale features via hierarchical upsampling and skip connections; and (4) an output head applying ReLU activation and adaptive average pooling to generate risk scores. The model was trained using fourfold cross-validation with a 75:25 train-validation split in each fold. Training employed a multi-class cross-entropy loss function, and validation accuracy was computed by comparing predicted and ground-truth labels. All experiments were conducted on an NVIDIA RTX 4090 GPU. Full implementation details are provided in Appendix 4.

### Data labeling

A two-step labeling strategy was employed to define the final ground truth for all patients by integrating overall survival time, arterial phase contrast-enhanced MRI features, mRECIST treatment response, and expert clinical judgment. Initially, patients were divided into two groups based on overall survival (OS): a short-term survival group (OS < 36 months) indicating rapid disease progression, and a long-term survival group (OS ≥ 36 months) suggesting better disease control. Within each survival group, further stratification was performed by systematically assessing imaging characteristics—including arterial phase enhancement patterns, internal enhancement heterogeneity or necrosis, tumor margin morphology, vascular invasion signs, and the presence of satellite nodules—alongside clinical evaluation. Treatment response according to the mRECIST was incorporated as an auxiliary reference to support risk refinement. Imaging data were independently reviewed by two board-certified radiologists, with final labels determined by consensus involving a senior interventional radiology expert. This classification process leveraged the Human-in-the-Loop (HITL) concept, integrating expert experience with quantitative data to enhance the accuracy and clinical relevance of AI training [42]. Patients were ultimately categorized into four risk groups: Class 0 (high-risk group), Class 1 (medium-to-high-risk group), Class 2 (low-to-medium-risk group), and Class 3 (low-risk group). A detailed description of the labeling procedure is provided in Appendix 5.

### AI model evaluation

We evaluated the model’s performance in stratifying HCC patients into four prognostic risk groups using fourfold cross-validation. Discriminative performance was assessed by AUC and classification accuracy. A one-vs-rest strategy was applied to compute class-specific AUCs (see Appendix 6 for formulas). To enhance interpretability, Grad-CAM +  + was used to generate activation maps highlighting image regions within the liver contributing to risk classification. Model generalizability was assessed across treatment subgroups (TACE alone and TACE + MWA) via Kaplan–Meier survival analysis. Prognostic factors for overall survival were identified through multivariate Cox regression. Comparative analyses were performed using mRECIST and five radiomics-based classifiers (Decision Tree, SVM, Random Forest, XGBoost, and Naive Bayes). Details of mRECIST implementation and the radiomics pipeline are provided in Appendix 7 and 8. To facilitate clinical application and further explore the clinical utility of the model, we conducted a post hoc multivariate analysis that integrated deep learning-derived risk scores with selected clinical variables and radiomic features—including age, sex, mRECIST response, and Sorafenib treatment—to support individualized overall survival prediction. A forest plot was generated to visualize the associated hazard ratios. Based on the results of this analysis, we constructed a nomogram to assist clinical decision-making. The nomogram development process is detailed in Appendix 9.

### Statistical analysis

Statistical analysis was performed using Python software, utilizing below libraries for different tasks. Specifically, Lifelines (https://lifelines.readthedocs.io/en/latest/) was employed for survival analysis, PyRadiomics (https://pyradiomics.readthedocs.io/en/latest/) for radiomics feature extraction, scikit-learn (https://scikit-learn.org/stable/) for machine learning tasks, and MONAI (https://monai.io/) as an open-source deep learning library for medical image analysis, along with the PyTorch deep learning framework (https://pytorch.org/). Categorical data were analyzed with the Pearson Chi-Square test. The AUCs were designed to assess the performance of models for survival classification. A p-value < 0.05 was considered statistically significant.

## Result

### Demographic and clinical characteristics of patients

In this study, we included a diverse cohort of real-world patients, as shown in Table 1. A total of 181 HCC patients were included in the retrospective cohort. The mean age (± standard deviation) of the entire retrospective cohort was 65.6 years ± 10.9, and 83.4% (n = 151) of the patients were men. Of the 181 patients, 96 (53.0%) was BCLC stage B and 52 (28.7%) was BCLC stage A. 18.2% (n = 33) of patients were at BCLC stage C.

**Table 1:**
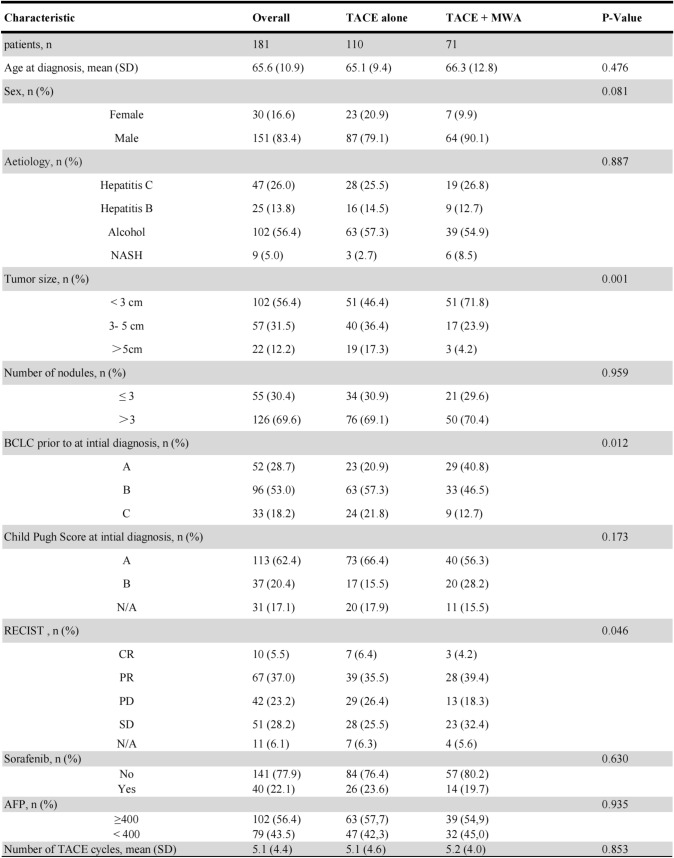
Baseline clinical characteristics of patients undergoing TACE treatment. The subgroup set were statistically analyzed using the Pearson Chi-Square test

The TACE monotherapy subgroup (n = 110) included 87 men (79.1%) and 23 women (20.9%), with a mean age of 65.1 ± 9.4 years. Most patients were BCLC stage B (57.3%), while 20.9% were BCLC stage A, and 21.8% were BCLC stage C. In the TACE + MWA subgroup (n = 71), the mean age was 66.3 ± 12.8 years, with 90.1% men. At diagnosis, 46.5% were BCLC stage B, 40.8% were BCLC stage A, and 12.9% were BCLC stage C. Univariate analysis showed significant differences in BCLC stages between the two cohorts (*p* = 0.012). Kaplan–Meier analysis (Appendix 10) showed significant differences in survival across BCLC stages in TACE monotherapy (*P* = 0.03), but not in the TACE + MWA group (*P* = 0.07), suggesting varied demographic characteristics among TACE-treated patients.

### Preprocessing segmentation for accurate tumor and liver delineation in HCC

**Fig. 3:**
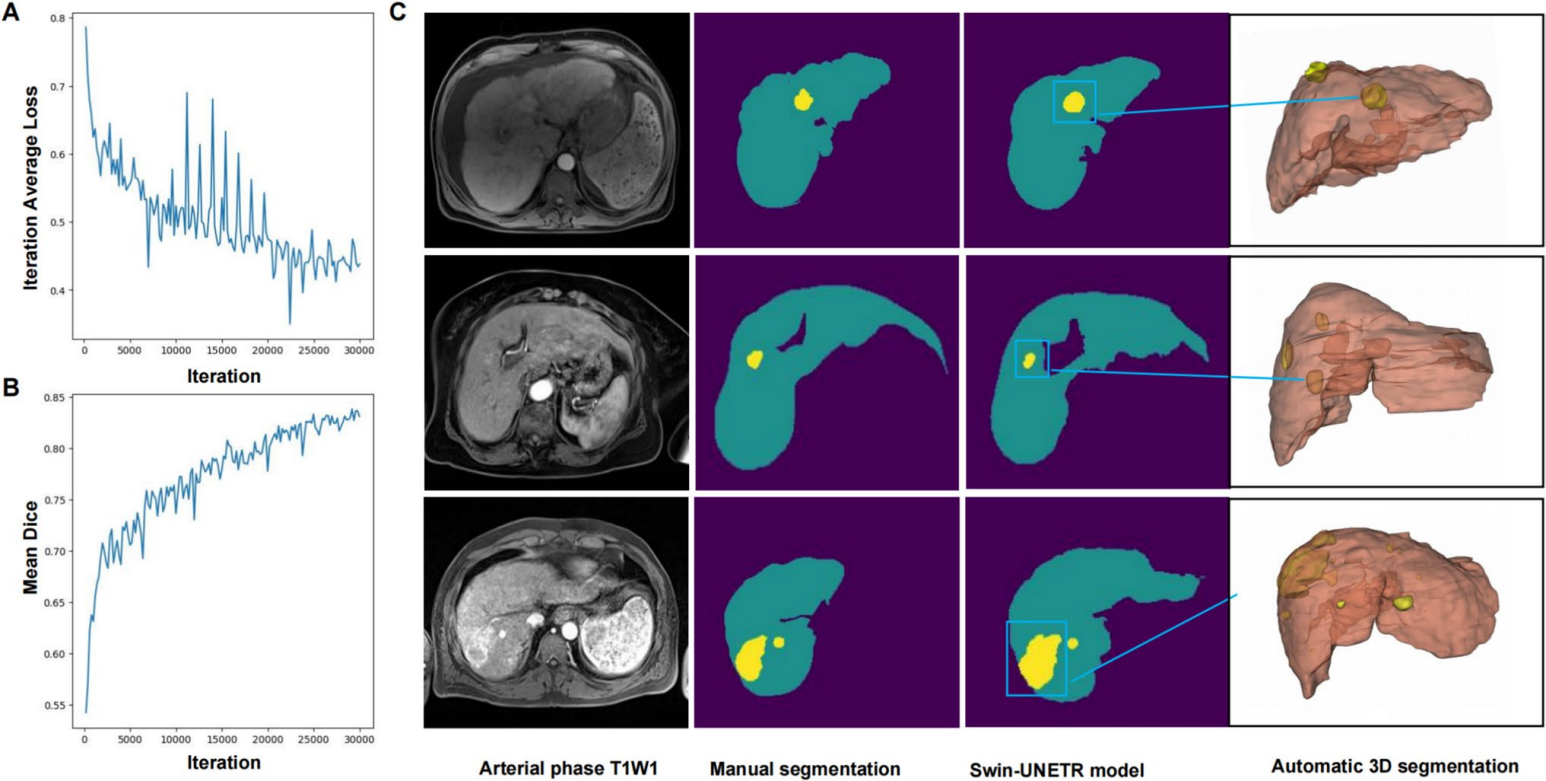
Three-dimensional arterial phase CE-MRI imaging segmentation using the deep learning model. **A** The average loss values over the number of iteration for training of Swin-UNETR segmentation model based with HCC patients arterial phase CE-MRI images. **B** Validation Dice curve, the highest dice coefficient for the image segmentation was 0.83. **C** The tumor segmentation results by the Swin-UNETR segmentation model and compared with manual label, the visualization result of segmented HCC tumors and liver background. Different color coverage areas represent different tumors: yellow for tumor, green for liver background

The training process of the ProgSwin-UNETR model over 30,000 iterations showed a steady decrease in average loss (Fig. 3A) and an improvement in the mean Dice score, stabilizing at 0.83 (Fig. 3B). Figure 3C presents a representative segmentation example, it is evident that the segmented image exhibits precise delineation. The segmented image accurately distinguishes between the liver background and tumor regions, effectively excluding the portal vein from the region of interest. The 3D segmentation results further demonstrate that the efficacy of the Swin-UNETR model in accurately segmenting multiple hepatocellular carcinoma lesions. All of the 3D segmentation results of tumors and the liver serve as regions of interest for radiomics and deep learning prognostic model research.

### Modeling process for developing the deep learning model

**Fig. 4:**
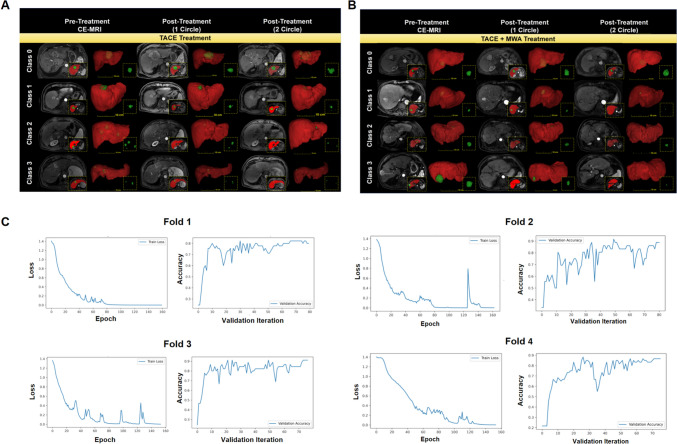
Model Training, and cross-validation for survival classification. Panel **A** demonstrates the samples from TACE-treated patients, while panel **B** includes samples from patients treated with TACE + MWA treatment. **A** and **B** illustrate the integration of arterial phase CE-MRI from three time points (pre-treatment and post-treatment at one and two circles) and the corresponding segmented masks for different survival classes (Class 0, Class 1, Class 2, Class 3). Each row represents a different survival class, showcasing the regions of interest overlaid on the arterial phase CE-MRI images, where red represents the liver background and green represents the tumor. **C** The training and validation performance for the ProgSwin-UNETR model across fourfold cross-validation. For each fold (Fold 1 to Fold 4), the left panels display the training loss curves, while the right panels show the validation accuracy curves, reflecting steady improvement with validation iterations

We constructed a deep learning model named ProgSwin-UNETR. ProgSwin-UNETR model was obtained from fourfold cross-validation training runs. In the training process, arterial phase CE-MRI images from the same patient at three clinical time points—prior to treatment initiation, after the first TACE cycle, and after the second TACE cycle—were combined with their corresponding segmented masks into a single three-channel training sample. Figure 4A and B demonstrates the integration of arterial phase CE-MRI data with segmentation masks for different survival classes (Class 0 to Class 3). Across all classes, the segmented regions of interest (ROIs) clearly highlight the tumor (green) and liver background (red), providing distinct spatial features for classification. In the output module, we had 4 output channels corresponding to survival classification (class0, class1, class2, class3). We applied the following hyperparameters: a batch size of 2, a learning rate of 0.001, a weight decay of 0.0001, and a region of interest (ROI) size of (64, 64, 64) during the sliding window inference process. Figure 4C shows the training and validation performance for the ProgSwin-UNETR model across fourfold cross-validation. For each fold (Fold 1 to Fold 4), the left panel shows the training loss curve as a function of epochs, indicating a steady decrease in loss over time, which stabilizes near the end of training (up to 160 epochs). The right panel presents the validation accuracy curve as a function of validation iterations, demonstrating the model’s increasing accuracy during the validation process. These results demonstrated the consistency and effectiveness of the deep learning model training process, with minimal overfitting observed across the folds.

### Classification effectiveness of the deep learning model in comparison with that of radiomics model

**Fig. 5:**
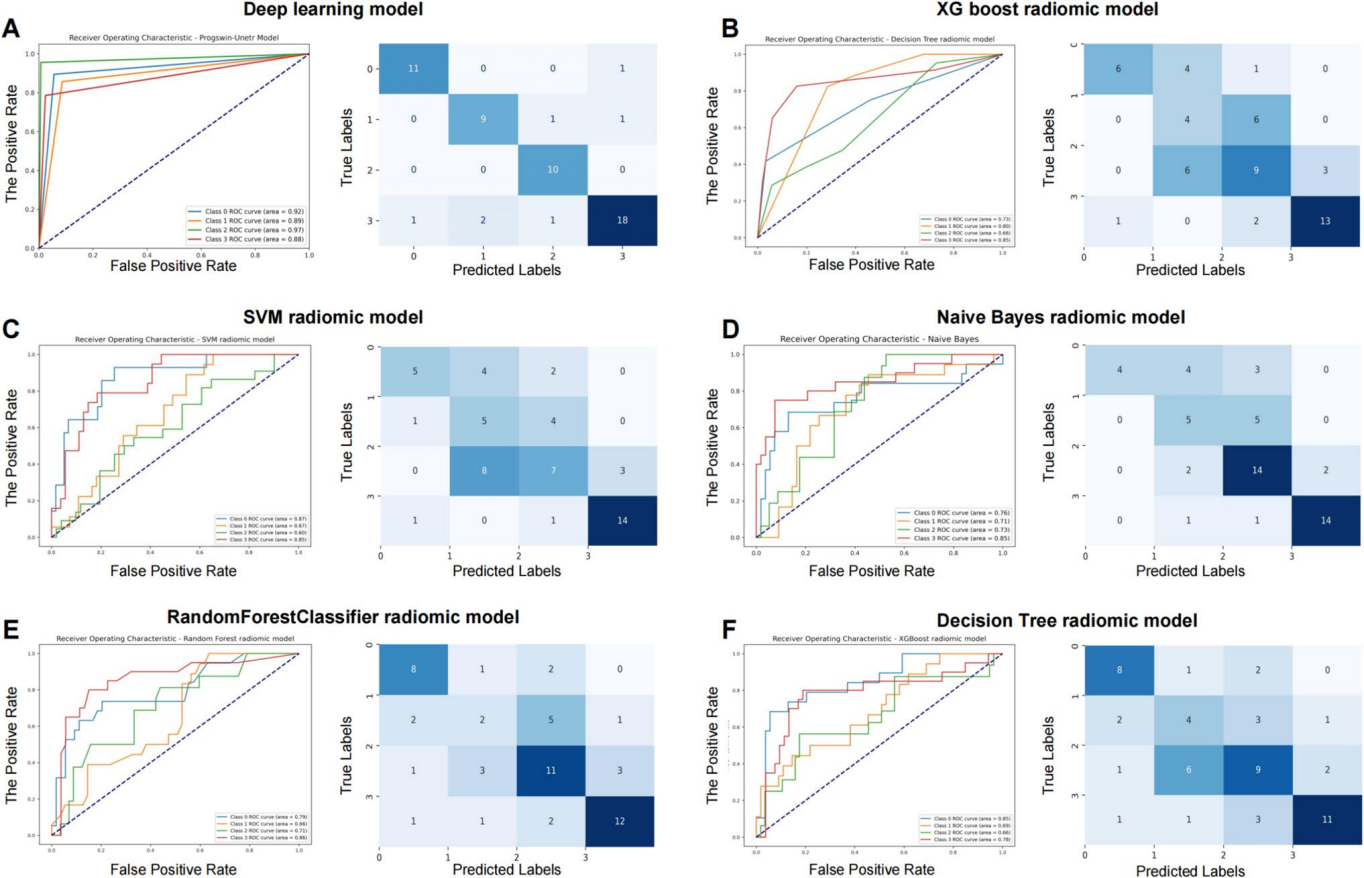
Receiver operating characteristic curves comparing the predictive performance of a deep learning model and various Radiomic models in risk group classification. The ProgSwin-UNETR model **A** demonstrated superior predictive performance across all risk groups, with AUC values of 0.92, 0.89, 0.97, and 0.88 for Class 0, Class 1, Class 2, and Class 3, respectively. Comparatively, the highest AUC values of specific risk groups among the radiomic models were observed in the Naive Bayes model **B** with 0.73 for Class 2, the Random Forest model **C** with 0.86 for Class 3, the SVM model **D** with 0.87 for Class 0, the Decision Tree model **E** with 0.80 for Class 1, indicating the ProgSwin-UNETR model’s overall superior performance

Figure 5A–F displays that the ProgSwin-UNETR deep learning model achieved a significantly higher average AUC (0.92; 95% CI: 0.90, 0.95) compared to the other five models in internal test cohort. The AUCs for the other five models in the internal test cohort were as follows: Naive Bayes radiomic model: 0.76 [95% CI: 0.66, 0.86], *P* < 0.05; Random forest classifier radiomic model: 0.75 [95% CI: 0.69, 0.82], *P* < 0.05; SVM radiomic model: 0.75 [95% CI: 0.65, 0.85], *P* < 0.05; Decision tree classifier radiomic model: 0.75 [95% CI: 0.66, 0.86], *P* < 0.05; XGBoost radiomic model: 0.74 [95% CI: 0.64, 0.85], *P* < 0.05. In addition, confusion matrices for the results of risk groups are presented in Fig. 5A–F. The corresponding accuracy and specificity were 0.87 and 0.94 for the internal test cohort for the ProgSwin-UNETR deep learning model. A detailed description of the models performance for predicting risk groups is outlined in Table 2.

**Table 2:**
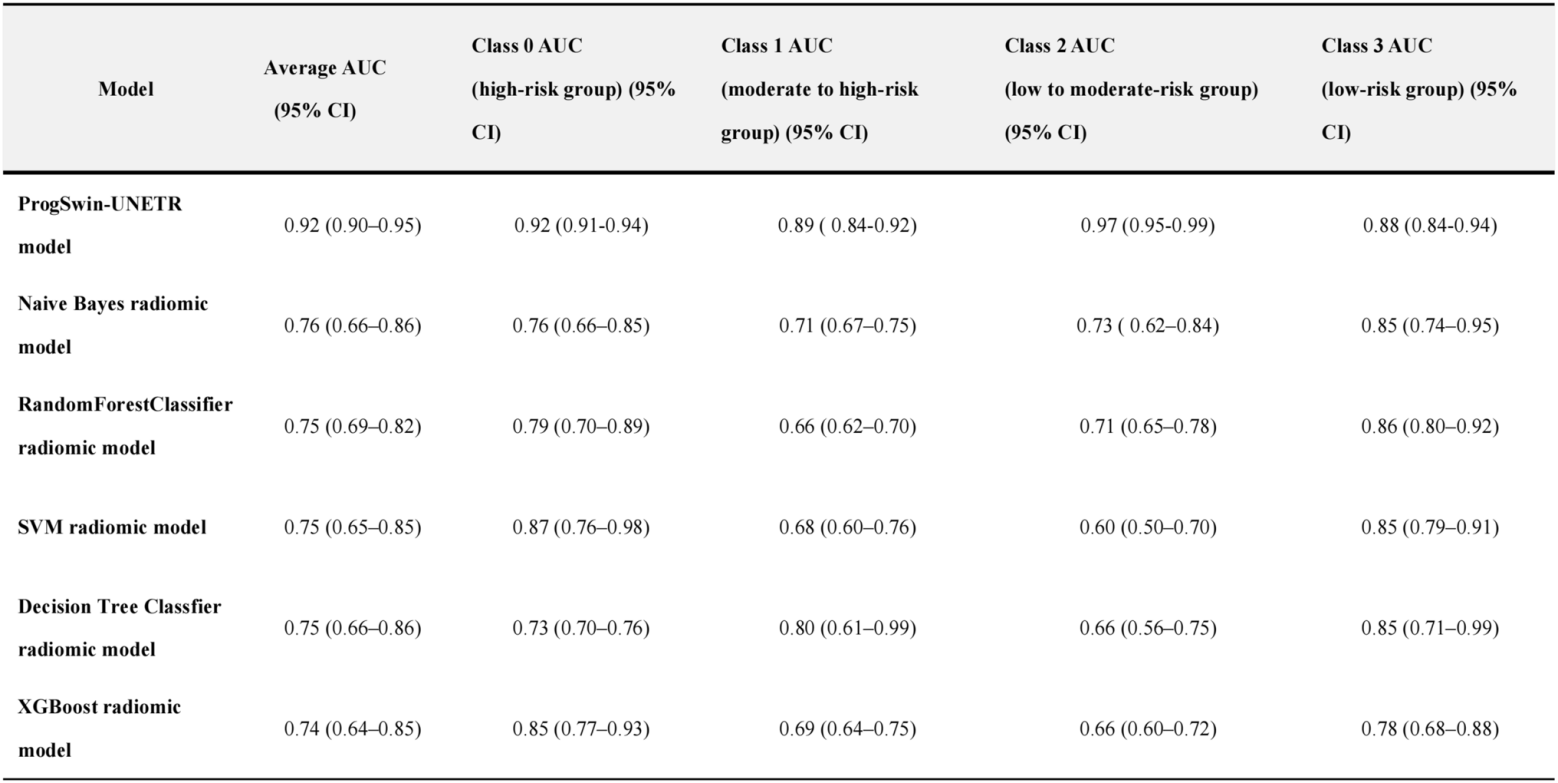
Average AUC and class-specific AUCs with 95% confidence intervals for deep leaning model and radiomic models

In this specific four-class prediction task, the deep learning model maintained a satisfactory predictive efficacy, consistently outperforming traditional radiomic models across all risk groups in terms of AUC and accuracy. In the high-risk group (Class 0) analyses, ProgSwin-UNETR achieved AUC of 0.92 (95% CI: 0.91–0.94), higher than the highest AUC of 0.87 (95% CI: 0.76–0.98) achieved by the SVM radiomic model among all the radiomic models. In the moderate to high-risk group (Class 1), ProgSwin-UNETR’s AUC was 0.89 (95% CI: 0.84–0.92), compared to the highest AUC in the radiomic model, which was 0.80 (95% CI: 0.61–0.99) from the Decision Tree Classifier radiomic model. For the low to moderate-risk group (Class 2), ProgSwin-UNETR achieved an AUC of 0.97 (95% CI: 0.95–0.99), surpassing the Naive Bayes radiomic model’s highest AUC of 0.73 (95% CI: 0.62–0.84). Lastly, in the low-risk group (Class 3), ProgSwin-UNETR’s AUC was 0.88 (95% CI: 0.84–0.94), higher than the highest AUC of 0.86 (95% CI: 0.80–0.92) from the Random Forest Classifier radiomic model.

### Predictive performance of deep learning model, radiomic model, and mRECIST criteria for individualized prognosis stratification

**Fig. 6:**
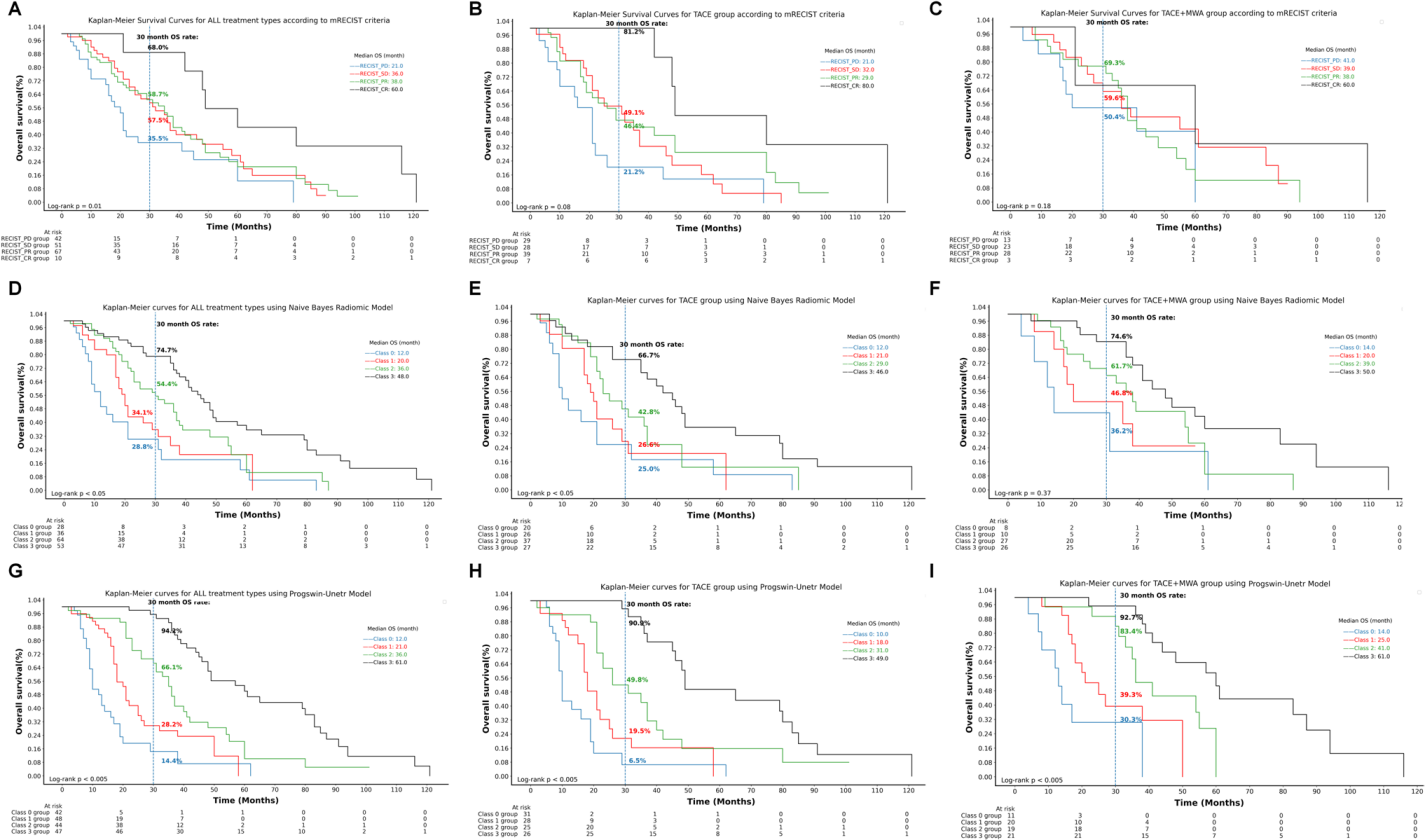
Kaplan–Meier plots showed the overall survival of HCC patients divided based on mRECIST criteria or predicted into four risk groups for prognosis using the Naive Bayes Radiomic Model and the ProgSwin-UNETR prognosis model. Patients were categorized according to the mRECIST criteria as follows: **A** those receiving all types of treatments (n = 181), **B** TACE monotherapy (n = 110), and **C** TACE + MWA combination therapy (n = 71). Patients are predicted into four risk groups based on the Naive Bayes Radiomic Model as follows: **D** those receiving all types of treatments, **E** TACE monotherapy, and **F** TACE + MWA combination therapy. Patients are predicted into four risk groups based on the ProgSwin-UNETR prognosis model as follows: **G** those receiving all types of treatments, **H** TACE monotherapy, and **I** TACE + MWA combination therapy. A vertical dashed line was used to mark an important evaluation point for midterm treatment efficacy in HCC patients

We used the cross-validation method with the ProgSwin-UNETR model to predict the whole cohort of patients into four risk groups. In the Kaplan–Meier survival analysis, we compared these risk groups with those categorized by the radiomic model and mRECIST criteria. As shown in Fig. 6A, the four different risk groups classified according to the mRECIST criteria exhibited significant differences in overall survival, with a log-rank test p-value of 0.01. In Fig. 6D, the Naive Bayes Radiomic model also demonstrated significant stratification of overall survival among the four risk groups (log-rank test p-value < 0.05). Similarly, Fig. 6G showed that the ProgSwin-UNETR model provided the highest significance in stratifying overall survival (log-rank test p-value < 0.005). All three models demonstrated significant statistical differences in stratifying the overall survival of patients within the entire cohort. A vertical dashed line at 30 months was used to mark an important evaluation point for midterm treatment efficacy in HCC patients, facilitating the comparison of overall survival rates across different response categories defined by various prognostic models.

Moreover, when analyzing two subgroups, the deep learning model exhibited superior performance. As shown in Fig. 6B and C, among the TACE monotherapy subgroup and TACE + MWA treatment subgroup, the four different risk groups classified according to the mRECIST criteria did not exhibit significant differences in overall survival in these two subgroups, with log-rank test p-values of 0.08 and 0.18, respectively. In Fig. 6E, the Naive Bayes Radiomic Model significantly stratified overall survival among the four risk groups (log-rank test p-value < 0.05), while in Fig. 6F, the model’s stratification was not significant, with a log-rank test p-value of 0.37. As shown in Fig. 6H and I, the ProgSwin-UNETR model demonstrated superior performance in stratifying patients into four risk groups. Both subgroups exhibited significant stratification of overall survival rates, with log-rank test p-values being less than 0.005.

### Multivariable cox regression analysis of the deep learning model, other prognostic models, and clinical characteristics

**Fig. 7:**
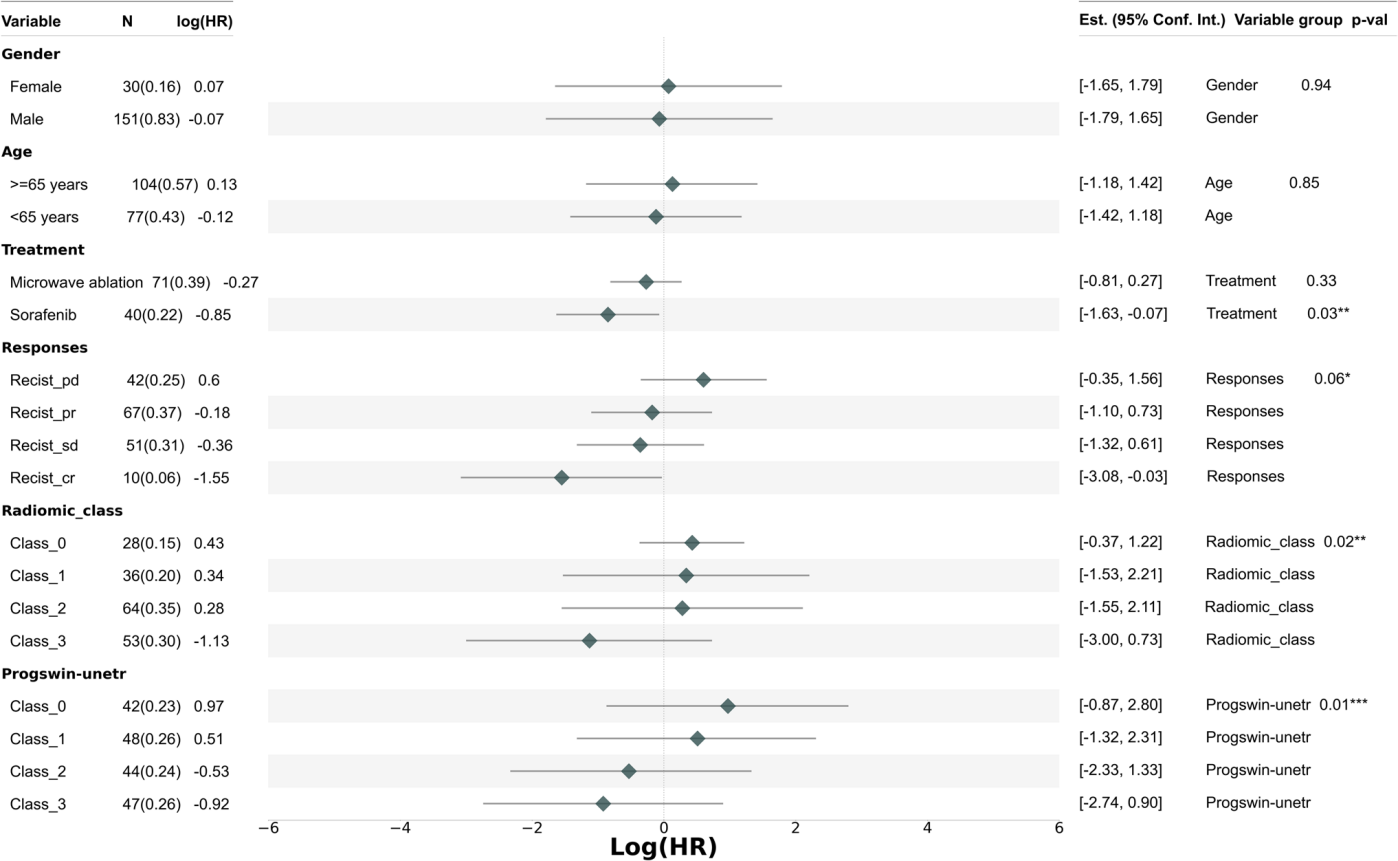
Multivariable Cox regression analysis to assess the impact of the deep learning (DL) model and clinical variables on the overall survival of HCC patients. The vertical dashed line indicates a log hazard ratio (logHR) of 0, with rhombuses representing the logHR values for each variable on the horizontal axis. Horizontal lines extending from the rhombuses denote the 95% confidence intervals (CI)

Multivariate Cox regression analysis showed that the ProgSwin-UNETR model not only accurately predicted patient outcomes but also stood as a robust independent prognostic factor (*p* = 0.01). Significantly, the AI model effectively stratified HCC patients into four distinct risk groups—Class 0 through Class 3—demonstrating a clear gradient in survival risks with Log(HR) values of 0.97, 0.51, −0.53, and −0.92, respectively. This stratification, visually depicted in Fig. 7, underscored our model’s precision in identifying varying levels of patient risk. Additionally, Sorafenib treatment, mRECIST, and the Radiomic score were identified as other significant prognostic factors for survival outcomes. The ProgSwin-UNETR model demonstrated excellent predictive performance for HCC patient survival, contributing to a concordance index (C-index) of 0.81 in the multivariate Cox regression analysis. Finally enhancing clinical application, we developed a nomogram that integrates these predictions with clinical and imaging, pinpointing patients with scores above 0.70 as high-risk. This crucial identification, illustrated in Appendix 11, enabled clinicians to consider more aggressive or alternative treatment strategies, potentially improving patient outcomes.

### Activation mapping of ProgSwin-UNETR model for different treatment subgroups

**Fig. 8:**
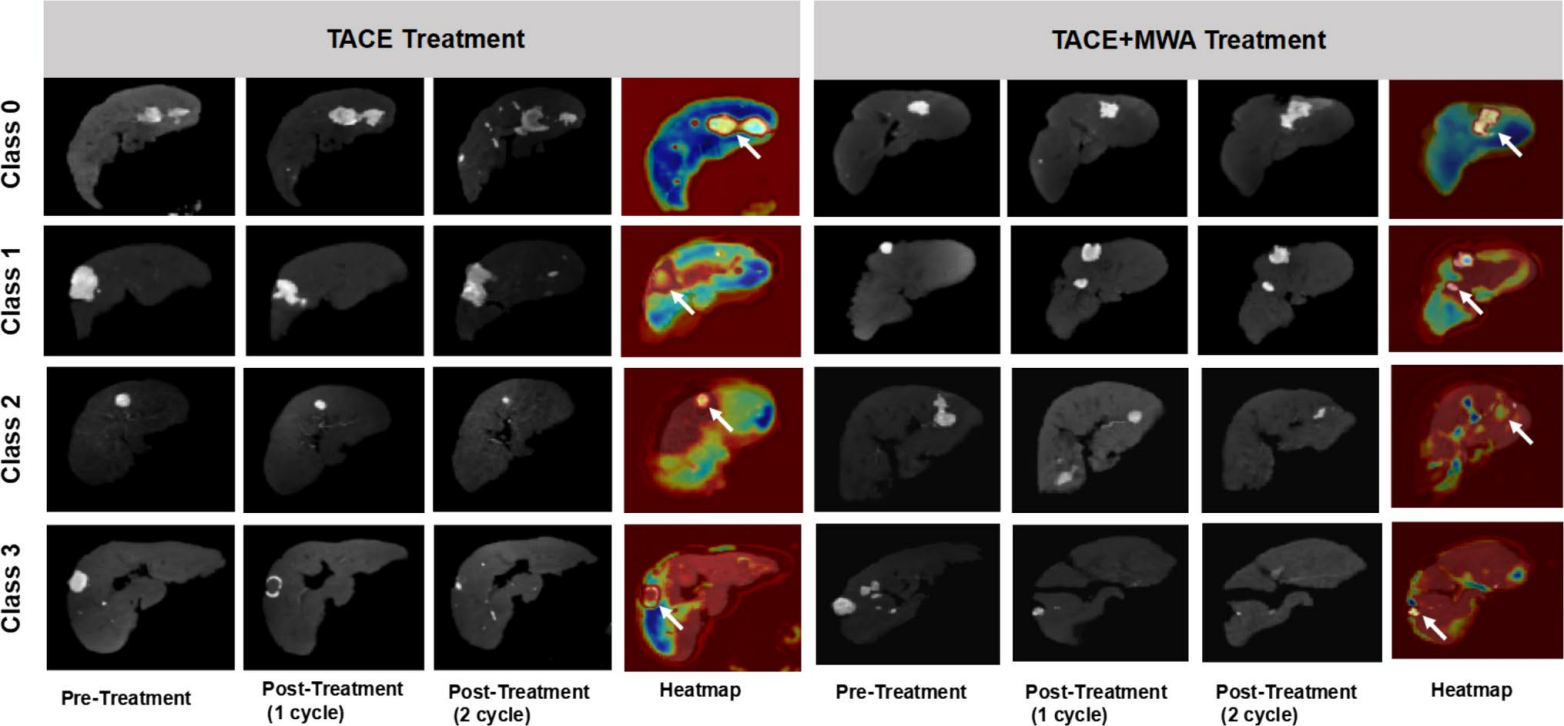
Activation mapping of ProgSwin-UNETR model for different treatment subgroups. There are eight representative cases. Comparison between two different treatment subgroups (TACE and TACE + MWA) using Grad-CAM +  + in different predicted risk groups. Each row represents activation maps of representative examples and arterial phase CE-MRI images at multiple time points: pre-treatment, post-treatment (1 cycle), post-treatment (2 cycles), and heatmap. The red areas in the activation maps indicate high relevance to patient prognosis. In the liver ROI heatmaps, areas with higher activation are represented in red, whereas blue indicates regions of lower activation. ROIs, regions of interest; Grad-CAM +  + , gradient-weighted class activation mapping +  + (a visualization technique to interpret deep learning models)

The activation mapping interpreted which areas in arterial phase CE-MRI were significantly connected with the prognosis of HCC patients. The Grad-CAM +  + visualization results for the TACE and TACE + MWA treatment groups across different risk classes (Class 0 to Class 3) are displayed (Fig. 8). Red signature in activation mapping indicated high relevance to prognosis of patients. In representative cases of two subgroups, Grad-CAM +  + heatmaps showed that the deep learning network primarily focused, which correspond to treatment-related changes of tumor and tumor metastasis in two subgroups. It proved that the model target the correct areas. Variations in the intensity of activation heatmaps were observed in the regions of interest (ROIs) corresponding to tumors. Residual or metastatic tumors displayed deeper color intensities in the heatmaps, indicating a more pronounced impact on prognostic outcomes. In Class 2 and Class 3 cases, the activation mapping further revealed that the deep learning network focused more extensively on the entire liver background region compared to Class 1 and Class 0. It indicated a correlation between positive prognosis and liver quality.

These findings demonstrated that the model had acquired the ability to extract valuable prognostic information from both the tumor area and the entire basal portion of the liver across multi-time-point images.

## Discussion

Personalized prognostic models support optimal management of HCC by enabling treatment strategies tailored to individual patient risk profiles. In this retrospective study, we investigated the performance of the ProgSwin-UNETR prognosis model by analyzing multi-time-point arterial phase CE-MRI of HCC patients. We implemented a Swin Transformer-based deep learning model, which achieved promising prognostic performance. Specifically, we utilized artificial intelligence to capture high-dimensional imaging features from arterial phase CE-MRI, incorporating long-term monitored individualized patient imaging information to predict prognosis. We also broadened the model’s applicability to HCC patients across different BCLC stages and treatment regimens, aiming to address real-world clinical complexity more effectively.

Monitoring tumor change in the course of treatment using imaging techniques is a standard step. The ability to extract important prognostic features from these medical images to predict overall survival is considered crucial for prognostic models. Traditional clinical scoring models for prognostic prediction in HCC have AUC values typically ranging from 0.53 to 0.79 [43–46]. Radiomics-based models have AUC values ranging from 0.74 to 0.81 [47]. The Swin Transformer-based deep learning model we established achieved a mean accuracy of 0.87 and demonstrated considerable performance with a mean AUC of 0.92 in the fourfold cross-validation set, offering a more precise, data-driven approach based on 3D image feature extraction for prognosis. This significant improvement highlighted our AI model’s superior capability in extracting crucial arterial phase CE-MRI features from the entire liver and integrating signatures across multiple time points during treatment. Beyond overall cohort performance, ProgSwin-UNETR demonstrated robust and clinically meaningful prognostic stratification across treatment subgroups. Kaplan–Meier analysis revealed that the model significantly differentiated four risk groups in both the TACE monotherapy and TACE + MWA subgroups (log-rank *p* < 0.005), where conventional mRECIST failed to reach statistical significance (*p* = 0.08 and 0.18, respectively). Similarly, while the radiomic-based model (Naive Bayes classifier) showed modest stratification in the TACE-only group (*p* < 0.05), it did not achieve significance in the TACE + MWA group (*p* = 0.37). These results underscore the superior generalizability and stability of our model across diverse treatment strategies.

ProgSwin-UNETR demonstrated practical value in supporting clinical management across multiple contexts. First, leveraging multi-time-point arterial phase CE-MRI, the model achieved consistently high AUCs (0.88–0.92) across four distinct risk strata, enabling fine-grained survival prediction and informing individualized treatment decisions. In the TACE subgroup, 31 of 110 patients were identified as high-risk, indicating limited benefit from repeated locoregional therapy and a potential need for alternative treatment strategies. Similarly, in the TACE + MWA subgroup, 11 of 71 patients were classified into the highest-risk group, with a median overall survival of only 14.0 months. For these individuals, continued locoregional intervention appears suboptimal, and early transition to systemic therapy would be more appropriate. Adjusting treatment in line with the AI model-predicted risk profiles might have improved their clinical outcomes. Although based on model inference and not yet validated in prospective trials, these findings support the potential clinical utility of AI-driven risk stratification for guiding timely treatment decisions in poor responders. Second, the interpretability of the AI model strengthened its clinical applicability. Grad-CAM +  + visualizations were used to identify key imaging regions that contributed to risk classification by computing the gradient of the target class with respect to a specific convolutional layer. In our heatmaps, high-risk cases consistently showed activation in tumor-dominant regions, whereas low-risk cases exhibited stronger signals in background liver tissue. This spatial differentiation provided an intuitive visual explanation of the model output, offering clinically meaningful insights and reinforcing physician confidence in AI-assisted decision-making. Third, the model demonstrated superior computational efficiency compared to conventional mRECIST-based assessment. Temporal features were automatically extracted and integrated within approximately 0.93 s per case, supporting rapid and reproducible prognostic evaluation while facilitating efficient resource allocation in routine clinical settings.

Furthermore, we conducted a post hoc multivariable Cox proportional hazards analysis to assess the feasibility of integrating AI-derived risk scores with clinical variables. C-index from post hoc Cox analysis was 0.81, suggesting good discrimination. Sorafenib treatment status remained an independent predictor of survival, underscoring the added prognostic value of clinical features beyond imaging-based data. Notably, among patients with similar AI risk scores, differences in treatment response—particularly with or without Sorafenib—were associated with markedly different survival outcomes. Patients with a cumulative nomogram score exceeding 0.70 were classified as high-risk. If the nomogram suggested that a patient had not received targeted therapy (e.g., sorafenib), initiation of such treatment could be considered. Conversely, if prior sorafenib use was documented, alternative systemic options—including immunotherapy or combination regimens—may be appropriate. This approach provides clinicians with a practical framework for translating risk scores into individualized prognostic estimates and informing evidence-based treatment decisions. Although not part of the core model, the nomogram illustrates how deep learning outputs can be integrated with clinical data to support risk stratification and guide treatment decisions for patients with poor prognosis.

However, our study had several limitations. First, this was a retrospective single-center study with a relatively small sample size. The lack of external validation might lead to bias. Although the use of cross-validation enhanced the robustness of the model to some extent, external validation on larger datasets is necessary to confirm the general feasibility of the proposed Swin-Transformer model before clinical application. Second, to reduce the computational burden, we excluded information outside the liver using computer masking technology. For some patients with ascites, this method might result in the loss of some useful information, thereby affecting the prediction accuracy of the model. Therefore, further optimization of the algorithm was needed to ensure that relevant information within and around the liver is retained while maintaining computational efficiency.

## Conclusions

Our study demonstrated that the ProgSwin-UNETR deep learning model offered robust predictive performance for HCC prognosis by effectively leveraging multi-time-point arterial phase CE-MRI data. The model’s ability to extract significant prognostic features has shown high accuracy and discriminative power across various risk groups, surpassing traditional radiomics models. Additionally, the integration of this model with key clinical variables enhanced its clinical utility, providing valuable insights for personalized patient management. Despite the promising results, our study had several limitations, including its retrospective nature, the need for external validation and the necessity for prospective study, which guided the path for our future work. Ultimately, our findings reinforced the potential of the ProgSwin-UNETR model as a transformative tool for improving prognostic assessments and clinical decision-making for patients with HCC.

## Appendix 1: Conventional TACE procedure and follow-up

Following the Seldinger technique via the femoral artery, a catheter pathway was established for abdominal arterial vascular imaging. Subsequently, the catheter was advanced through the abdominal aorta, positioned within the common hepatic artery. It was then navigated through the segmental and subsegmental branches of the liver to the farthest segmental artery capable of accommodating the tumor. An ultra-selective approach was employed for the injection of chemotherapeutic-Lipiodol-mix plus/minus In cases where both hepatic lobes were affected, priority was given to treating the hepatic lobe with a higher tumor burden. During the treatment procedure, a suspension of Mitomycin C (Medac®, Hamburg, Germany) and embolic agent Lipiodol (Guerbet GmbH, France) in a 1:2 ratio was initially administered. The maximum allowable dose of Mitomycin C was 8 mg/m2. A maximum of 5 ml of Lipiodol was administered in each session.A treatment decision-making period consisted of three consecutive TACE treatments. In case of new lesions or disease progression during the follow-up period, our multidisciplinary tumor committee would reassess the patient to explore alternative treatment options.

## Appendix 2: Microwave ablation procedure

In the TACE + MWA combined treatment, TACE was used as a downstaging procedure to reduce the size and number of lesions to meet the criteria for ablation. The microwave ablation procedure was planned based on the most recent contrast-enhanced MRI scans available for each patient. The target lesion was identified, the optimal placement for the ablation antenna was determined, and the entry site was marked on the patient’s skin using radiopaque markers. Throughout the procedure, patients were continuously monitored using blood pressure measurements, pulse oximetry, and electrocardiography. Prior to the procedure, patients received a combination of sedative and analgesic medications, including diazepam (Diazepam-ratiopharm®, ratiopharm GmbH) at a dose of 0.1–0.2 mg/kg body weight and piritramide (Piritramid-hameln®, Hameln Pharma Plus GmbH) at a dose of 0.2 mg/kg body weight. The ablation procedures were performed using the Covidien Emprint™ microwave ablation (MWA) system equipped with Thermosphere™ Technology. Under computed tomography (CT) guidance, the ablation antenna was inserted into the lesion using a 128-slice multi-detector CT scanner (Somatom Definition AS, Siemens) with the following settings: 5 mm fade-in, 30 mAs, 120 kV, 5 mm slice thickness, and real-time tube current modulation (CARE Dose 4D, Siemens). This precise and carefully controlled approach ensured safe penetration and advancement of the antenna into the target lesion while minimizing the risk of injury to surrounding tissues. Once proper placement of the antenna was confirmed, the ablation process was initiated. The MWA was conducted in three steps with rising output powers (45–60W, 65–80 W, and 85–100 W). Towards the end of treatment, the puncture site of the inserted electrode was coagulated during retraction to prevent tumor seeding or possible bleeding. The patients were subsequently observed and monitored for the next 12 h in the hospital. Serial CT scans were performed throughout the procedure to monitor the ablation and to detect any early complications. Adjustments to the antenna’s position were made when necessary to achieve optimal therapeutic outcomes. Following sufficient ablation time, the antenna was carefully removed, and the entry tract was coagulated to reduce the risk of inadvertent tumor cell dissemination.

## Appendix 3: Imaging segmentation process of the arterial phase CE-MRI

We initially collected Arterial Phase CE-MRI images at different time points, including before treatment, after the first treatment session, and after the second treatment session. Firstly, two especially trained radiologists (LLY) and (HL) conducted the initial independent segmentation with the following steps: manually delineating the three-dimensional regions of interest (ROIs) for both the liver background and the tumor on the patient’s contrast-enhanced Arterial Phase CE-MRI images while preserving regions with ambiguous boundaries. Segmentations were controlled by a board-certified radiologist (TV) with more than 20 years of experience. Subsequently, we employed the initially segmented images for training the Swin-UNETR artificial intelligence image segmentation model to obtain a preliminary model. Following that, we underwent multiple rounds of iteration, continuously adjusting and optimizing the model to ensure the highest segmentation accuracy. Ultimately, we applied the model with the highest segmentation accuracy to all images to achieve high-quality segmentation.

## Appendix 4: Technical details of model construction

The study developed the ProgSwin-UNETR deep learning network to independently predict prognostic risk stratification of HCC patients. Before inputting the images into the network, the original images were cropped**.** Specifically, we first obtained the 3D mask of liver cancer and its liver background using segmentation techniques. The segmentation process to obtain the 3D mask of the liver and cancer region can be described as:

1 $$\left( {M_{1} \left( {x,y,z} \right),\;M_{2} \left( {x,y,z} \right)} \right) \, = {\text{ Segmentation}}\left( {I\left( {x,y,z} \right)} \right)$$

where *M₁*(*x, y, z*) and *M₂*(*x, y, z*) represent the 3D segmentation masks for the two targets, where *M₁*(*x, y, z*) corresponds to the first target (e.g., liver background) and *M₂*(*x, y, z*) corresponds to the second target (e.g., tumor). *I*(*x, y, z*) denotes the original intensity value of the image at the position (*x, y, z*).

In the validation Dice curve, the highest Dice coefficient for image segmentation was 0.83.

2 $$\text{Dice}(M,G)=\frac{2\times (\mid {M}_{1}\cap {G}_{1}\mid +\mid {M}_{2}\cap {G}_{2}\mid ) }{\mid {M}_{1}\mid +\mid {G}_{1}\mid +\mid {M}_{2}\mid +\mid {G}_{2}\mid }$$

where *M*_*1*_ ∩ *G*_*1*_and *M*_*2*_ ∩ *G*_*2*_ represent the intersections of the predicted and ground truth segmentations for the liver and tumor, respectively, and *M*_*1*_, *G*_*1*_, *M*_*2*_ and *G*_*2*_ represent the sizes of the corresponding predicted and ground truth regions. The overall Dice coefficient takes into account the combined performance of both the liver and tumor segmentations.

The network architecture consisted of 4 main components. The first component included an image enhancement module, which processed 3D medical images with segmentation masks, enhanced target regions, resized them to (128, 128, 128), stacked three channels, and output a 4D one-hot encoded vector.

3 $${I}_{enhanced =}=\text{stack}({I}_{t1}\odot M,{I}_{t2}\odot M,{I}_{t3}\odot M)$$

where Each* I*_*t1*,_ *I*_*t2*,_ *I*_*t3*_ are 3D images volume representing the imaging data for a specific time point. stack(): Combines the three processed images into a single input with three channels. ⊙: Combines the mask and images, keeping only the important regions. *I*_enhanced_ is the final input tensor, with dimensions (3,128,128,128).

The next two components involved the use of a Swin-Transformer in the encoder, which efficiently extracted image features through self-attention mechanisms and hierarchical feature representations.

4 $$F = {\text{SwinEncoder}}\left( {I_{{{\text{enhanced}}}} } \right)$$

where *F* represents the feature set extracted from the enhanced image. The decoder leveraged the UNet architecture to accurately map these features onto the final output layer.

5 $$O = {\text{UNetDecoder}}\left( F \right)$$

where *O* is the final output from the decoder. The final component applied Rectified Linear Unit (ReLU) activation functions and adaptive average pooling to 3D input tensors, output the probabilities of the target categories.

6 $$P = {\text{ReLU}}\left( {{\text{AdaptivePooling}}\left( O \right)} \right)$$

where P represents the output probabilities for each class. Furthermore, we used four-fold cross-validation schemes with a ratio of 75:25 to get training set and validation set as a result of the limited quantity of data available. During model training, we used the nn.crossentropyloss() loss function.

7 $$\text{Loss}=-\frac{1}{N} \sum_{i=1}^{N} \sum_{c = 1}^{C}{t}_{i, c} \cdot \text{log}({p}_{i, c})$$

where *t*_*i, c*_ is the true label for sample *i* in class *c*, for the correct class *y*_*i*_*, t*_*i,c*_ = 1; for other classes, t_i,c_ = 0*. p*_*i,c*_ is the predicted probability that sample* i* belongs to class *c*. *y*_*i*_ is the true class label for sample *i*; and *N* is the total number of samples. During model validation, accuracy was calculated by using the `torch.eq` function to determine the consistency between the predicted and actual classes.

8 $$Accuracy=\frac{1}{N} \sum_{i=1}^{N} 1({P}_{i}={y}_{i})$$

where *P*_*i*_ is the predicted class for the i-th sample (obtained through ReLU and AdaptivePooling processing), and* y*_*i*_ is the true class label for the i-th sample. The indicator function 1(*P*_*i*_ = *y*_*i*_) equals 1 if the predicted class *P*_*i*_ matches the true class *y*_*i*_; otherwise, it is 0. The model was run on an NVIDIA RTX 4090 graphics card.

## Appendix 5: The process of the labeling

The final labels divide all samples into four risk groups: low-risk, low-to-medium-risk, medium-to-high-risk, and high-risk. To optimize patient risk stratification and prognosis assessment, we first categorized patients based on survival time, followed by further refinement into four risk groups according to expert judgment. This process integrates machine learning predictions with clinical expertise, incorporating the "Human-in-the-Loop" (HITL) approach proposed by Hunter, D. J. Through this methodology, the efficiency of the AI system is enhanced by combining it with the expertise of clinicians, demonstrating the potential of integrating machine learning and clinical experience for optimizing risk assessment in HCC patients undergoing TACE. This ensures that prognosis evaluation is informed by both quantitative data and clinical judgment.

Initially, patients were categorized into two groups based on their survival time:

- *Short-term survival patients* Patients with a survival time of less than 36 months, indicating rapid disease progression.
- *Long-term survival patients* Patients with a survival time exceeding 36 months, indicating better disease control and prolonged survival.
- Subsequently, based on imaging data and expert clinical judgment, the stratification was further refined. In this process, imaging evaluation systematically considered key features closely related to tumor biology and prognosis, including arterial phase enhancement patterns (typical or atypical), internal enhancement heterogeneity or extent of necrosis, tumor margin morphology (smooth or irregular), imaging signs of vascular invasion, and the presence of satellite nodules or peritumoral enhancement. Additionally, treatment response evaluated according to the modified Response Evaluation Criteria in Solid Tumors (mRECIST) was incorporated as a complementary reference to support risk stratification. Two radiologists (LLY and HL) assessed the patients’ imaging data, with all results reviewed and validated by a board-certified interventional radiologist (TV) with more than 20 years of experience.

The specific classification strategy is as follows:

*Class_0 (High-risk group)* Short-term survival patients at high risk, as indicated by rapid disease progression observed on imaging, with poor prognosis.

*Class_1 (Medium-to-high-risk group)* Short-term survival patients at low risk, with milder disease features on imaging and relatively stable prognosis despite shorter survival time.

*Class_2 (Low-to-medium-risk group)* Long-term survival patients at high risk, showing more severe disease features on imaging despite longer survival, with an uncertain prognosis.

*Class_3 (Low-risk group)* Long-term survival patients at low risk, with well-controlled disease as shown on imaging, suggesting longer survival and favorable prognosis.

## Appendix 6: Evaluation metrics of the AI model

We evaluated the performance of the AI model in identifying HCC prognostic risk groups using assessment metrics such as the area under the curve (AUC) and accuracy, based on fourfold cross-validation. The AUC can be calculated individually using a "one-vs-rest" approach.

9 $${\text{AUC}}_{c}={\int }_{0}^{1}{\text{TPR}}_{c}(x)d({\text{FPR}}_{c}\left(x\right))$$

Where class *c* (where c = 0, 1, 2, 3), TPR*c*(*x*) is the True Positive Rate for class* c*, representing the proportion of correctly identified positives for class *c*. FPR*c*(*x*) is the False Positive Rate for class *c*, representing the proportion of other classes incorrectly identified as class *c*. We employed the Grad-CAM +  + algorithm to generate activation mapping of liver areas correlated with survival classification.

10 $${L}_{\text{Grad}-\text{CAM}++}^{c}=\text{ReLU }\left(\sum_{k}{\alpha }_{k}^{c}{A}^{K}\right)$$

The AI model’s generalizability was assessed across different treatment subgroups using the Kaplan–Meier method, including TACE monotherapy and TACE combined with microwave ablation (TACE + MWA).

11 $$\widehat{S}(t)=\prod_{{\text{t}}_{\text{i}}\le \text{t}}\left(1-\frac{{d}_{i}}{{n}_{i}}\right)$$

where $\widehat{S}(t)$ is the estimated survival probability at time *t*, *t*_*i*_ represents the overall survival time (OS),

*d*_*i*_ is the number of events at time* t*_*i*_, *n*_*i*_ is the number of patients at risk just before time* t*_*i*_. Multivariate Cox proportional hazards regression model analyses were performed to identify potential prognostic factors for OS.

12 $${\text{h}}\left( {{\text{t}}{\mid }{\text{X}}} \right) = {\text{h}}_{0} \left( {\text{t}} \right){\text{exp}}\left( {\beta 1{\text{X}}_{{1}} + \beta_{2} {\text{X}}_{{2}} + \cdots + \beta_{p} {\text{X}}_{p} } \right)$$

where h(t|X) is the hazard function at time* t* given covariates *X*, h_0_(*t*) is the baseline hazard function (the hazard when all X_i_ = 0), X = (X_1_,X_2_,…,X_p_) represents the covariates, *Β* = (*β*_*1*_*, β*_*2*_*,…, β*_*p*_) are the coefficients associated with each covariate, estimated from the data.

## Appendix 7: The classification of tumor response according to mRECIST criteria

According to mRECIST criteria, the corresponding responses included: (1) Complete Response (CR), indicating complete tumor disappearance; (2) Partial Response (PR), defined as a reduction of at least 30% in the minimum diameter of visible target lesions during the arterial phase; (3) Progressive Disease (PD), characterized by an increase of at least 20% in the minimum diameter of visible target lesions; and (4) Stable Disease (SD), indicating neither PR nor PD.

## Appendix 8: The process of the radiomic analysis

We utilized an open-source package (https://github.com/AIMHarvard/pyradiomics/blob/master/examples/exampleSettings/exampleMR_3mm.yaml) to extract radiomic features. The feature definitions in PyRadiomics adhere to the IBSI consensus. From seven feature classes, we extracted all original standard features, including shape-based (3D), first-order statistics, gray-level co-occurrence matrix (GLCM), gray-level run length matrix (GLRLM), gray-level size zone matrix (GLSZM), gray-level dependence matrix (GLDM), and neighboring gray tone difference matrix (NGTDM), resulting in 1132 features per volume of interest (VOI) and sequence. Prior to feature dimension reduction, we standardized all extracted radiomic features to ensure uniform variance in the data. We employed parallel feature selection strategies, which included recursive feature addition (RFA), recursive feature elimination with cross-validation (RFECV), as well as machine learning models such as random forests and gradient boosting trees. We selected a total of 181 radiomics features from the ROI of Arterial Phase CE-MRI imaging based on the first post-TACE Arterial Phase CE-MRI imaging. As shown in the Appendix 5, the features underwent further optimization and refinement using the least absolute shrinkage and selection operator (LASSO) logistic regression algorithm, which was fine-tuned through GridSearchCV. During this process, we selected non-zero features to further optimize and streamline the feature set. Radiomics feature selection via the LASSO regression algorithm, a total of 8 features were deemed most relevant. Using Pearson correlation coefficient to investigate the correlation among the 8 texture features. The final 7 features were visualized in three dimensions through Principal Component Analysis (PCA). In the feature modeling phase, we employed five distinct machine learning models for feature analysis, including random forest classifier, XGBoost, support vector machine, Bayesian classifier, and decision tree. Subsequently, to evaluate the performance of our proposed model and validate its generalization ability to unseen data, we adopted a strategy of splitting the dataset into training and testing sets. We utilized the `train_test_split` function to partition the dataset, with the training set comprising 75% of the total samples and the testing set comprising 25%. We utilized data splitting functions to construct queues for calculating the area under the curve (AUC) and generating ROC curves to evaluate the model’s performance.

Radiomics feature selection via the LASSO regression algorithm. (A) Feature selection using LASSO regression to predict survival time. LASSO regression showed λ = 0.1 when the error of the model was minimized. (B) showed that 8 features selection was performed by fivefold cross-validation with the criterion of minimum deviance. (C) The confusion matrix illustrates the correlation between 8 features, with each cell containing a correlation coefficient representing the relationship between respective features. There is a significant correlation between Original_shape_SurfaceArea feature and wavelet_Lhh_glszm_graylevelnonuniformity feature (r = 0.93). The correlation among other features is not significant. The final 7 features were selected finally. (D) The 3D PCA scatter plot depicts the distribution of the 7 radiomic feature dataset across the first three principal components, with each distinct group represented by a different color. It illustrates PCA results, where four main clusters are evident in three-dimensional space: one yellow and one red cluster on the left and right, respectively, with a green cluster in between. Notably, no distinct orange cluster was observed, suggesting potential underlying patterns within the extracted image features and failure to entirely partition the data points into four distinct categories.

## Appendix 9: Construction of a prognostic nomogram integrating deep learning risk scores, clinical variables, and radiomics features for individualized survival prediction in HCC

To enhance clinical interpretability of our deep learning model for HCC, we constructed a post hoc prognostic nomogram integrating DL-derived risk scores, clinical covariates, and radiomics features for individualized overall survival (OS) prediction. Three categories of variables were included: (1) risk scores output by the DL model trained on arterial-phase contrast-enhanced MRI; (2) clinical variables (age at diagnosis, sex, sorafenib treatment status, and RECIST response); and (3) radiomics-derived features extracted from tumor regions. Continuous variables were standardized; categorical variables were one-hot encoded. Missing values were imputed or excluded based on availability and clinical relevance. We fitted a multivariable Cox proportional hazards model to estimate hazard ratios (HRs) and 95% confidence intervals (CIs). Variables with significant prognostic value (*p* < 0.05) were retained for the final model. The proportional hazards assumption was assessed using Schoenfeld residuals. Coefficients from the final Cox model were mapped to point scales in a nomogram. Each patient’s total point score was computed and used to estimate individualized survival probabilities. A data-driven cutoff (e.g., total score > 0.70) was defined to stratify patients into low- and high-risk groups. The latter were characterized by poorer prognosis and potentially reduced treatment response, indicating a need for intensified therapeutic strategies. The nomogram was visualized using standard plotting tools to facilitate interpretation. It serves as a graphical interface for translating AI-derived predictions into actionable survival estimates, supporting precision oncology decision-making in HCC management.

## Appendix 10: Kaplan–meier plots of overall survival and progression-free survival stratified by BCLC stage for the entire cohort, TACE monotherapy, and TACE + MWA treatment groups

(A) Kaplan–Meier plots of overall survival for whole cohort patients who are straitified according to BCLC stage, including patient from all treatment types stratified by the three BCLC stages. The median survival time among all patients was 36 months, There is a significant difference in HCC patients for different BCLC stages in whole cohort (*P* = 0.01, log-rank test). (B) Patients stratified by the BCLC stages from TACE monotherapy showed significant differences across various BCLC stages (*P* = 0.03, log-rank test). (C) Patients undergoing TACE + MWA treatment did not show significant differences across various BCLC stages (*P* = 0.07, log-rank test). (D, E, F) Progression-free survival (PFS) curves revealed that the trends observed in PFS survival were consistent with those seen in overall survival.

## Appendix 11: Nomogram for predicting high-risk treatment-resistant patients

This nomogram was developed to visually and quantitatively predict high-risk treatment-resistant patients based on several variables, including gender, age at diagnosis, Sorafenib treatment, mRECIST score, radiomic score, and ProgSwin-UNETR score. High-risk treatment-resistant patients are those with a poor prognosis under standard treatments and are less likely to respond to therapy.

In the nomogram for overall survival (OS), each independent prognostic marker is assigned a value that can be plotted on the points axis. The cumulative points from all markers are then used to determine the patient’s risk level by referencing the overall points axis and drawing a line down to the survival axis. Patients with a cumulative score above 0.70 are classified as high-risk treatment-resistant. These patients, having poorer prognoses, should be considered for alternative treatment strategies in order to improve outcomes.

## Abbreviations

**Arterial Phase CE-MRI**: Arterial phase contrast-enhanced magnetic resonance imaging
**TACE**: Transarterial chemoembolization
**HCC**: Hepatocellular carcinoma
**AI**: Artificial intelligence
**mRECIST**: Modified response evaluation criteria in solid tumors
**KM**: Kaplan–meier
**BCLC**: Barcelona clinic liver cancer
**MWA**: Microwave ablation
**DL**: Deep learning

## Declarations

### Supplementary Information

Supplementary information associated with this article has been submitted.
